# Management of endocrine immune-related adverse events of immune checkpoint inhibitors: an updated review

**DOI:** 10.1530/EC-20-0342

**Published:** 2020-09-16

**Authors:** Maria Stelmachowska-Banaś, Izabella Czajka-Oraniec

**Affiliations:** 1 Department of Endocrinology, The Centre of Postgraduate Medical Education, Warsaw, Polska, Poland

**Keywords:** immunotherapy, immune checkpoint inhibitors, CTLA-4, PD-1, PD-L1, hypophysitis, thyroid disorders, diabetes mellitus, adrenal insufficiency, hypoparathyroidism

## Abstract

Immune checkpoint inhibitors (ICIs) belong to a new group of anticancer drugs targeting T-cell proteins involved in the activation of immune response toward malignancies. Their introduction into clinical practice was a milestone in modern cancer treatment. However, the significant advantage of ICIs over conventional chemotherapy in terms of therapeutic efficacy is accompanied by new challenges related to specific side effects. ICI-induced immune system activation could lead to the loss of self-tolerance, presenting as autoimmune inflammation and dysfunction of various tissues and organs. Thus, the typical side effects of ICIs include immune-related adverse events (irAEs), among which endocrine irAEs, affecting numerous endocrine glands, have been commonly recognized. This review aimed to outline the current knowledge regarding ICI-induced endocrine disorders from a clinical perspective. We present updated information on the incidence and clinical development of ICI-induced endocrinopathies, including the most frequent thyroiditis and hypophysitis, the rarely observed insulin-dependent diabetes mellitus and primary adrenal insufficiency, and the recently described cases of hypoparathyroidism and lipodystrophy. Practical guidelines for monitoring, diagnosis, and treatment of ICI-related endocrine toxicities are also offered. Rising awareness of endocrine irAEs among oncologists, endocrinologists, and other health professionals caring for patients receiving ICIs could contribute to better safety and efficacy. As immunotherapy becomes widespread and approved for new types of malignancies, increased incidences of endocrine irAEs are expected in the future.

## Introduction

Over the past several years, immunotherapy with immune checkpoint inhibitors (ICIs) has become an effective treatment of many malignancies. Immune checkpoints are molecules on the surface of immune cells involved in the regulation of the immune response, while ICIs are monoclonal antibodies directed against certain immune checkpoints, such as cytotoxic T-lymphocyte-associated protein 4 (CTLA-4) (ipilimumab) and programmed death 1 (PD-1) (nivolumab, pembrolizumab) and its ligand (PD-L1) (atezolizumab, avelumab), resulting in T-cell activation and anti-tumor activity. However, immune checkpoints also play a crucial role in maintaining immunological self-tolerance and preventing autoimmune disorders. Interfering with this mechanism can cause immune-related adverse events (irAEs) presenting as autoimmune disorders affecting numerous organs in the body. Endocrinopathies are among the most common irAEs associated with ICI therapy. Thyroid disorders (TDs), typically associated with anti-PD-1 antibodies and hypophysitis (IH) commonly related to anti-CTLA-4 therapy, are the most frequent endocrine toxicities. ICI-induced insulin-deficient diabetes mellitus (DM) and primary adrenal insufficiency (PAI), though rare, can be life-threatening if not promptly diagnosed and treated. The combination of anti-CTLA-4 and anti-PD-1 treatment has generally been associated with the highest incidence and severity of ICI-induced endocrinopathies (1). Considering the growing population of patients receiving immunotherapy with increasingly broad indications, rising number of patients with endocrine irAEs should be expected in the near future. Additionally, new endocrine irAEs, such as primary hypoparathyroidism, acquired generalized lipodystrophy, and autoimmune polyglandular syndrome, have been described recently (2). Some endocrinopathies, such as thyrotoxicosis, may be transient and resolve spontaneously after only monitoring or symptom control, whereas others, such as central adrenal insufficiency, primary hypothyroidism, or insulin-deficient diabetes, are persistent and require appropriate lifelong hormonal replacement. While non-endocrine irAEs often require cessation of immunotherapy and usually resolve with immunosuppressive therapy, that is, high-doses of glucocorticoids (GCSs) (3, 4), endocrine irAEs generally do not require cessation of ICI-treatment and rarely require high-dose GCS therapy, although long-term management may be required when persistent (5, 6). Interestingly, the presence of certain irAEs, such as TDs and IH, is associated with significant improvement in clinical outcomes in cancer patients, that is, longer median progression free survival (PFS) and overall survival (OS) (7, 8, 9). On the other hand, there could be a potential negative effect of high-dose GCSs on ICI treatment efficacy (10). Endocrinologists will often be requested to co-manage patients with endocrine irAEs and should be familiar with details specific to ICI-induced endocrinopathies. Given the increasing use of ICIs, cooperation between oncologist and endocrinologist is crucial in the management of patients receiving the same. Establishing an appropriate diagnosis and adequate management may improve the prognosis of oncological patients suffering from immunotherapy-induced endocrinopathy.

This review covers current practices related to the diagnosis and treatment of ICI therapy-associated endocrinopathies, with insights into their pathophysiology and clinical manifestations to improve outcomes of oncological patients.

## Immune checkpoint inhibitor-induced hypophysitis

### Definition

ICI-induced hypophysitis is a clinical condition associated with ICI use that is characterized by pituitary gland inflammation and consequent dysfunction (hypopituitarism), with some cases exhibiting enlargement causing a mass effect.

### Epidemiology

Pituitary inflammation as a primary condition (e.g. lymphocytic hypophysitis) or secondary to systemic diseases, such as systemic lupus erythematosus or sarcoidosis, is a very rare entity (11). The estimated annual incidence of hypophysitis is approximately one case per 9 million individuals (12). In recent years, however, an increase in the incidence of IH has been observed due to the introduction and extended use of ICIs.

IH has been described as the most frequent endocrine irAE associated with ipilimumab administration. However, broad variations in incidence rates have been reported (0–17%) (13). This could be attributed to differences in the diagnostic criteria used, changes in the clinical awareness of IH, differences in the frequency of hormonal tests, and improved clinical recognition (14). Currently, routine hormonal testing allows for the diagnosis of endocrinopathies also among asymptomatic patients (8).

Several authors have reported high IH frequencies (11–13%) (15, 16) and relative risk for all-grade IH as high as 22.03 (17). However, according to the latest reviews and meta-analyses IH incidences are lower but they notably depend on the type of ICI used and the treatment regimen (1, 18, 19, 20, 21, 22, 23). Combination therapy has been associated with the highest estimated incidence of IH, ranging from 7.7 to 10.5%, remarkably higher than monotherapy with anti-CTLA-4 (1.8–5.6%) or anti-PD-1 drugs (0.3–1.1%) (1, 18, 19, 20, 21, 22, 23). These data are summarized in Table 1. Thus far, only few cases of pituitary involvement during avelumab (24) and atezolizumab (25, 26) treatment have been published. To sum up, patients on combination therapy (ipilimumab plus nivolumab) were significantly more likely to develop IH than those receiving ipilimumab in monotherapy (odds ratio OR = 2.2). In turn, patients treated with anti-PD-1 drugs had significantly lower risk for any grade IH compared to those on anti-CTLA-4 (OR = 0.29) (1).

**Table 1:** Estimated incidences of endocrine irAEs during ICIs treatment based on the recent meta-analyses.

Endocrine irAEs	ICIs – drug groups and treatment regimens			References
	Anti-CTLA-4	Anti-PD-1/anti PD-L1	Combined treatment Anti-CTLA-4 + anti-PD-1	
Hypophysitis				
Any-grade	4.13% (Ipi)	0.31% (Niv)	10.4% (Ipi + Niv)	Almutairi *et al.* (18)
		0.66% (Pem)	10.46% (Ipi + Pem)	
	4.53%	<1%	7.68%	Lu *et al.* (19)
	3.30%	NA	NA	Xu *et al.* (20)
	1.8% (Tre)	0.5% (Niv)	8.8% (Ipi + Niv)	de Filette *et al.* (21)
	5.6% (Ipi)	1.1% (Pem)	10.5% (Ipi + Pem)	
	3.80%	1.1% (anti-PD-1)	8.0% (Ipi + Niv)	Barroso-Sousa *et al.* (1)
	NA	0.30%	NA	Baxi *et al.* (22)
	NA	0.85%	NA	Wang *et al.* (23)
	**1.8–5.6%**	**0.3–1.1%**	**7.68–10.5%**	
Serious-grade (≥3)	2.06% (Ipi)	0.15% (anti-PD-1)	1.96% (Ipi + Pem)	Almutairi *et al.* (18)
			2.36% (Ipi + Niv)	
	0.78%	<0.1%	1.66%	Lu *et al.* (19)
	1.70%	NA	NA	Xu *et al.* (20)
	NA	0.20%	NA	Baxi *et al.* (22)
	NA	0.60%	NA	Wang *et al.* (23)
	**0.78–2.06%**	**<0.1–0.6%**	**1.66–2.36%**	
Hypothyroidism				
Any-grade	2.84% (Ipi)	7.02% (Niv)	16.34% (Ipi + Pem)	Almutairi *et al.* (18)
		8.34% (Pem)	16.39% (Ipi + Niv)	
	2.50%	NA	NA	Xu *et al.* (20)
	3.8% (Ipi)	4.7–6.0% (anti-PD-L1)	10.2% (Tre + anti-PD-L1)	de Filette *et al.* (21)
		8.0–8.5% (anti-PD-1)	15.1–16.4% (Ipi + anti-PD-1)	
	3.80%	3.9% (anti-PD-L1)	13.2% (Ipi + Niv)	Barroso-Sousa *et al.* (1)
		7.0% (anti-PD-1)		
	NA	5.60%	NA	Baxi *et al.* (22)
	NA	7.00%	NA	Wang *et al.* (23)
	**2.5–3.8%**	**3.9–8.5%**	**10.2–16.4%**	
Serious-grade (≥3)	0% (Ipi)	0% (anti-PD-1)	0% (Ipi + Pem)	Almutairi *et al.* (18)
			0.08% (Ipi + Niv)	
	0.40%	NA	NA	Xu *et al.* (20)
	NA	0.20%	NA	Baxi *et al.* (22)
	NA	0.80%	NA	Wang *et al.* (23)
	**0–0.4%**	**0–0.8%**	**0–0.08%**	
Thyrotoxicosis (hyperthyroidism)				
Any-grade	0.9% (Ipi)	3.01% (Niv)	10.16% (Ipi + Niv)	Almutairi *et al.* (18)
		3.34% (Pem)	11.11% (Ipi + Pem)	
	0.20%	NA	NA	Xu *et al.* (20)
	1.4% (Ipi)	2.3% (Ave)	9.4% (Ipi + Niv)	de Filette *et al.* (21)
		2.8–3.7% (anti-PD-1)	10.4% (Ipi + Pem)	
	1.70%	0.6% (anti-PD-L1)	8.0% (Ipi + Niv)	Barroso-Sousa *et al.* (1)
		3.2% (anti-PD-1)		
	NA	3.50%	NA	Wang *et al.* (23)
	**0.2–5.2%**	**0.6–3.7%**	**8.0–11.1%**	
Serious-grade (≥3)	0.1% (Ipi)	0% (anti-PD-1)	0.66% (Ipi + Niv)	Almutairi *et al.* (18)
			1.31% (Ipi + Pem)	
	0.20%	NA	NA	Xu *et al.* (20)
	NA	0.47%	NA	Wang *et al.* (23)
	**0.1–0.2%**	**0–0.47%**	**0.66–1.31%**	
DM				
Any-grade	0% (Ipi)	2.4% (Pem)	1–2%	Almutairi *et al.* (18)
	0.52% (Ipi)	4.86% (anti-PD-1)	3.37%	Lu *et al.* (19)
		0.81% (anti-PD-L1)		
	0%	2% (Niv)	2%	de Filette *et al.* (21)
		0.4% (Pem)		
		1.4% (Atz)		
	NA	0.20%	NA	Barroso-Sousa *et al.* (1)
	**0–0.52%**	**0.2–4.86%**	**2–3.37%**	
Serious-grade (≥3)	0% (Ipi)	0.05% (Pem)	1.06–1.96%	Almutairi *et al.* (18)
	0.06% (Ipi)	0.49% (anti-PD-1)	0.47%	Lu *et al.* (19)
		0% (anti-PD-L1)		
	NA	0.10%	NA	Barroso-Sousa *et al.* (1)
	**0–0.06%**	**0–0.49%**	**0.47–1.96%**	
PAI				
Any-grade	1.4% (Ipi)	2% (Niv)	5.2–7.6%	de Filette *et al.* (21)
		0.8% (Pem)		
		1.1% (Ave)		
	0.7% (Ipi)	0.70%	4.20%	Barroso-Sousa *et al.* (1)
	**0.7–1.4%**	**0.7–2%**	**4.2–7.6%**	
Serious-grade (≥3)	**0.20%**	**0.20%**	**NA**	Barroso-Sousa *et al.* (1)

Atz, atezolizumab; Ave, avelumab; CTLA-4, cytotoxic T-lymphocyte associated protein-4; DM, diabetes mellitus; ICIs, immune checkpoint inhibitors; Ipi, ipilimumab; irAEs, immune-related adverse events; NA, not available; Niv, nivolumab; PAI, primary adrenal insufficiency; PD-1, programmed cell death-1; PD-L1, programmed cell death ligand-1; Pem, pembrolizumab; Tre, tremelimumab.

### Pathogenesis

The marked differences in the incidences of anti-CTLA-4 antibody-induced and anti-PD-1 or anti-PD-L1 antibody-induced hypophysitis may be attributed to functional differences in the process of T cell activation (27), and the expression of CTLA-4 in human pituitary gland cells that may be targeted by an anti-CTLA-4 antibody (28). Nonetheless, the pathophysiological mechanisms have not been completely understood.

### Risk factors

Although some studies have shown a positive association between ipilimumab dose and the incidence of IH (29), another wide analysis suggested that no endocrine irAEs seem to be dose dependent (30). The association between IH, gender and age needs to be further investigated because the data are not consistent. Some authors have reported higher frequencies of IH in older males (over the age of 60 years) (2, 14, 15). This is in contrast to primary hypophysitis, which is found mostly in younger females (12). However, these data should be taken cautiously because the gender and age may not be an independent risk factor for IH (8, 31) but they could be a result of the demographic characteristics of patients receiving ICIs (32, 33).

### Clinical presentation and diagnosis

#### Onset of immune checkpoint inhibitor-induced hypophysitis

Patients receiving ipilimumab (9.3 weeks, interquartile range (IR) 7.2–11.1) or combination therapy (12.5 weeks, IR 7.4–18.6) had a significantly shorter median IH onset time compared to those receiving anti-PD1 antibodies (25.8 weeks, IR 18.4–44.0) (34). Therefore, most cases developed ipilimumab-induced IH after the third drug infusion (16, 35), while anti-PD-1/PD-L1-induced IH might be expected months after treatment initiation (25, 36).

#### Clinical characteristics

Symptoms of IH can be relatively non-specific and progress insidiously, making diagnosis challenging for clinicians. The most common presenting symptoms of IH include headaches, fatigue, generalized weakness, nausea, appetite loss, cold intolerance, and dizziness, with rare occurrences of confusion or visual disturbance (31, 37). Milder symptoms may be overlooked or attributed to the underlying neoplastic disease. Early diagnosis and proper treatment are remarkably important given that the disease can progress to a potentially life-threatening state, mainly due to secondary adrenal insufficiency (SAI). Dehydration and hypotension, sometimes severe and refractory, have been observed on clinical examination (38), while hyponatremia and hypoglycemia have been frequently present (37, 39).

#### Diagnostic procedures

Hormonal test results showing hypopituitarism are crucial for the diagnosis of IH. Majority of patients, especially those treated with ipilimumab, have multiple (three or two) hormone deficiencies, usually affecting corticotropin, thyrotropin, and gonadotropin secretion (2, 3, 31), although isolated anterior pituitary hormone deficiency can be present as well (2). Isolated corticotroph insufficiency is the most common dysfunction and it is irreversible in the majority of cases (40). Moreover, hyperprolactinemia or very low prolactin levels have been less frequent (31), while posterior pituitary involvement has been very rare, with only a few cases of diabetes insipidus (2, 24, 41) or the syndrome of inappropriate anti-diuretic hormone (SIADH) (42) following ICI treatment having been reported. Recently, two cases of ICI-induced transient adrenocorticotropic hormone (ACTH)-dependent hypercortisolemia, with (43) or without (44) clinical features of Cushing syndrome, followed by SAI due to destructive IH, have been described.

**Figure 1:**
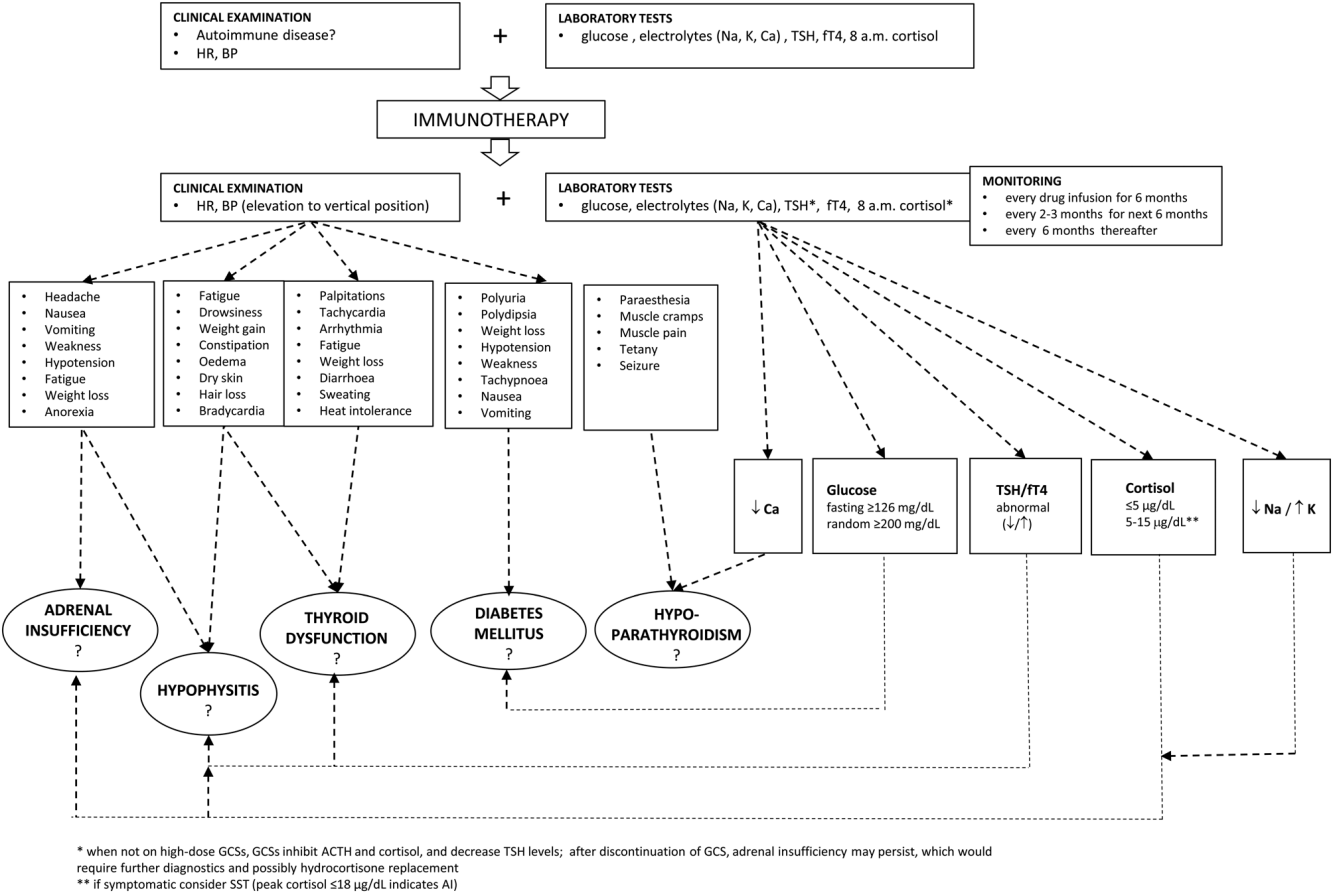
The proposed algorithm for the screening of endocrine disorders during immune checkpoint inhibitor treatment. ACTH, adrenocorticotropic hormone; BP, blood pressure; Ca, calcium; fT4, free thyroxine; GCSs, glucocorticoids; HR, heart rate; K, potassium; Na, sodium; SST, short Synacthen test; TSH, thyroid stimulating hormone.

Given the high risk of developing endocrine irAEs, such as IH, among patients receiving ICI, screening for endocrinopathies before treatment initiation followed by regular monitoring, preferably at every infusion for at least 6 months and less frequently thereafter, is generally recommended (45). Initial tests should include fasting glucose, electrolytes, thyroid stimulating hormone (TSH) and free T4 (fT4), and early morning cortisol levels (Fig. 1). Patients with low cortisol levels (≤5 µg/dL) should be assessed for plasma ACTH. Determining testosterone, luteinizing hormone (LH), and follicle-stimulating hormone (FSH) levels in males, FSH levels in postmenopausal women, and estradiol, LH, and FSH levels in premenopausal women with irregular menstruations (3, 33, 45) can also be useful. Previous or current treatment with steroids, which could substantially alter cortisol and ACTH results and decrease TSH level, should be considered.

**Figure 2:**
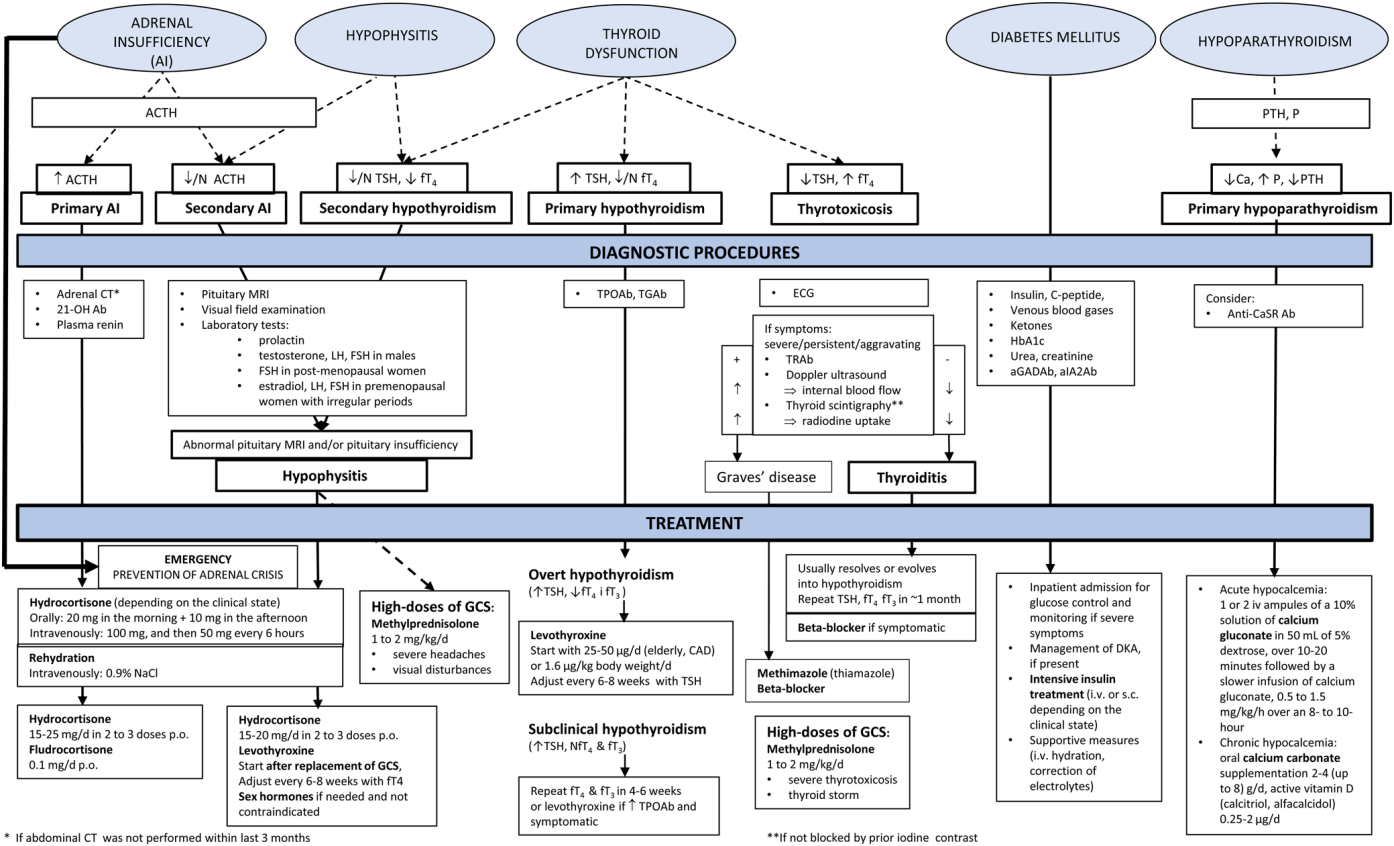
The proposed algorithm for the diagnostic procedures and management of endocrine immune-related adverse events. 21-OH Ab, 21-hydroxylase autoantibodies; ACTH, adrenocorticotropic hormone; aGADAb, anti-glutamic acid decarboxylase antibodies; AI, adrenal insufficiency; aIA2Ab, anti-tyrosine phosphatase IA2 antibodies; Anti-CaSRAb, anti-calcium sensing receptor antibodies; Ca, calcium; CAD, coronary artery disease; CT, computed tomography; DKA, diabetic ketoacidosis; ECG, electrocardiogram; FSH, follicle-stimulating hormone; fT3, free triiodothyronine; fT4, free thyroxine; GCSs, glucocorticoids; HbA1c, hemoglobin A1c; K, potassium; LH, luteinizing hormone; MRI, magnetic resonance imaging; Na, sodium; P, phosphorus; PTH, parathyroid hormone; SST, short Synacthen test; TGAb, thyroglobulin antibodies; TPOAb, thyroid peroxidase antibodies; TRAb, thyroid-stimulating hormone receptor antibodies; TSH, thyroid stimulating hormone.

Upon clinical suspicion of IH, new laboratory tests, including electrolytes, TSH, fT4, cortisol, ACTH, prolactin, and the aforementioned sex hormones, are necessary (Fig. 2). Accordingly, a decrease in TSH levels is one of the earliest changes in hormonal levels that may appear prior to IH diagnosis and cortisol decrease (46). A Synacthen (cosyntropin, 1–24 ACTH) stimulation test might be performed when basal cortisol measurements are inconclusive (Fig. 1). It should be considered that the results may be falsely reassuring in the early phase of SAI, when the adrenal glands may still respond normally to the stimulation (33).

**Figure 3:**
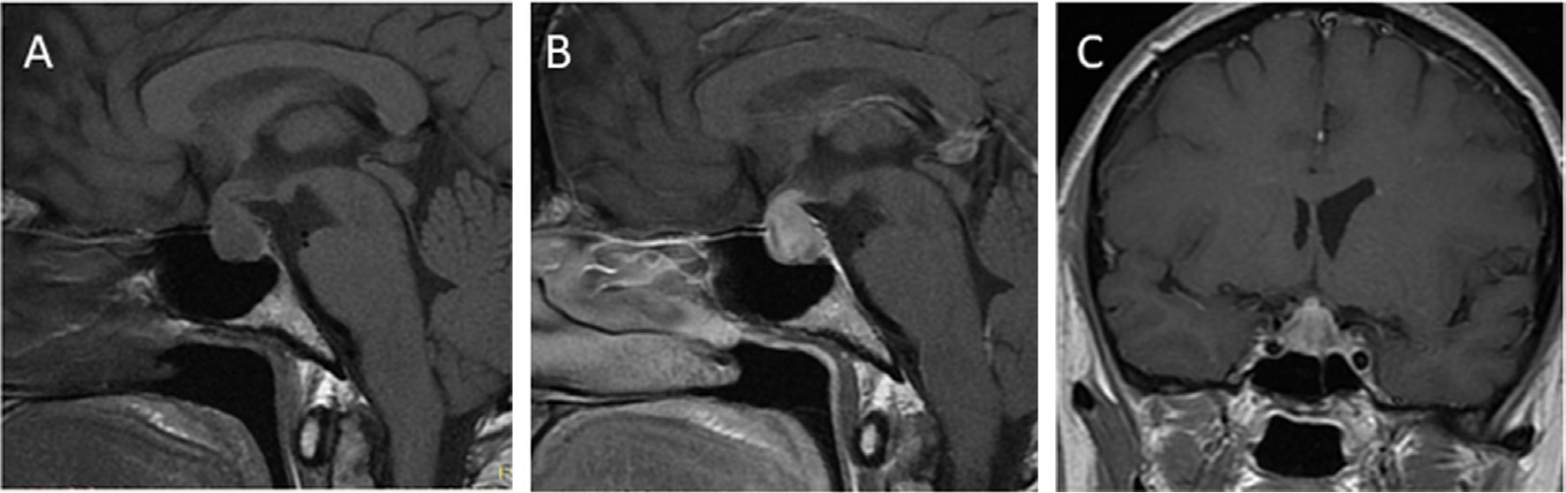
Pituitary MRI: sagittal T1-weighted images before (A) and after gadolinium enhancement (B) and a coronal T1-weighted image after gadolinium enhancement (C) in a 56-year-old female patient with melanoma who developed ICI-induced hypophysitis after the third dose of ipilimumab.

When IH is suspected and/or hypopituitarism is present, the proceeding diagnostic tests should incorporate brain/pituitary MRI (MRI). During the acute phase of IH, MRI findings frequently indicate pituitary gland abnormalities, with enlargement, stalk thickening, and homogeneous or heterogenous contrast enhancement, especially in patients treated with anti-CTLA-4 (Fig. 3) (16, 35). In sporadic cases, a mass effect on the optic chiasm can be observed (47, 48). In some cases, an empty sella has been described on diagnosis (36), which may develop spontaneously or during treatment (16, 21). However, given that up to one third of patients may have no abnormalities on MRI (2), especially those treated with anti-PD-1/anti-PD-L1 (49), a diagnosis of IH cannot be overlooked despite normal imaging results (45). MRI should not delay hormonal workup and treatment initiation, which mostly depend on clinical presentation and demonstration of hypopituitarism (6, 50). On the other hand, pituitary MRI abnormalities can precede the development of pituitary deficiency (3). Hence, in cases where MRI changes suggestive of IH present with normal blood tests results, closer monitoring of hormone levels should be implemented (45). Brain MRI is also important to exclude symptomatic brain or pituitary metastases, abscess, or pituitary apoplexy (51). Metastases to the pituitary gland are more prone to develop in the posterior lobe, frequently causing diabetes insipidus which is extremely rare in IH (48).

The proposed confirmatory criteria for IH include ≥1 pituitary hormone deficiency (TSH or ACTH deficiency required) combined with an MRI abnormality or ≥2 pituitary hormone deficiencies in the presence of headache and other symptoms (3). However, it is important to be aware of the differences depending on the type of ICI used, as it has been shown that IH due to anti-PD1/PD-L1 drugs often appears as an isolated SAI with normal MRI results (34, 49, 52). Thus, in anti-PD-1/anti-PD-L1 treated patients, diagnosis of IH is mainly based on clinical symptoms (often fatigue and hyponatremia) and presence of a new pituitary insufficiency (usually isolated ACTH deficiency). Confirmation of IH diagnosis through histologic examination of a surgical biopsy is not indicated unless other pituitary pathologies, such as metastasis, is strongly suspected (45).

### Management

In most cases, IH results in permanent hypopituitarism regardless of treatment attempts with high-dose GCSs (4, 33, 53). While recovery of corticotroph function is unusual, that of thyroproph and gonadotroph varies from 6% to 64% and from 12% to 57%, respectively (33). Given that high-dose GCSs did not seem to reverse hypopituitarism, they are recommended only in selected patients with adrenal crisis, severe headaches, and visual disturbances due to significant pituitary enlargement and optic chiasm compression (3, 33, 45, 50, 53). In the majority of patients, long-term hormonal replacement is sufficient.

SAI should be initially diagnosed and treated. Patients with mild or moderate symptoms (grade 1 or 2, respectively, on the Common Terminology Criteria for Adverse Events (CTCAE) scale, version 5.0) and low serum cortisol levels (≤5 µg/dL) should begin oral hydrocortisone (HC) replacement. However, a substantial group of patients may present with severe or life-threating symptoms of IH (grade 3 or 4, respectively) (1). They should be assumed to have acute cortisol deficiency until proven otherwise, and started on GCSs as in adrenal crisis (bolus injection of 100 mg of HC IV followed by 200 mg of HC delivered over 24 h in continuous infusion or in 50 mg injections every 6 h) before the serum cortisol result available (53, 54). Extensive rehydration with isotonic saline is routinely required. Significant improvement is usually observed within the first 24 h following correction of cortisol deficiency. After improvement of clinical symptoms and biochemical parameters, oral HC treatment should be continued at a dose of 60 mg/24 h in three administrations and progressively reduced to a replacement dose of 15–20 mg/24 h (45). Equivalent doses of longer-acting GCSs could be used for example, 3.75-5 mg of prednisone/prednisolone or 0.5–0.75 mg of dexamethasone (55). When pretreatment cortisol level is above 15 µg/dL, cortisol deficiency may be excluded, and GCS treatment discontinued.

Treatment for thyrotropin deficiency is less urgent and should be initiated when fT4 levels are below normal limits, but only after HC replacement has been implemented to avoid precipitating an adrenal crisis (33, 56). We recommend starting with a low dose of levothyroxine (25–50 μg/day), especially in older patients and those with coronary artery disease, and adjusting the dose afterwards as necessary (57). The target fT4 level should be in the mid to upper half of the reference range (55). Gonadotropin deficiency can be corrected if the gonadal axis did not recover after 3 months and no contraindications are present. Rare cases of diabetes insipidus may require desmopressin replacement. Measurement of growth hormone and IGF-1 levels is unnecessary given that replacement is contraindicated in patients with active cancer. However, further reevaluation to assess pituitary function recovery and patient well-being are regularly needed. Follow-up pituitary MRI is recommended at 3 months in patients with pituitary enlargement during initial examination (45).

Immunotherapy should be withheld during the acute, symptomatic phase of IH but can be continued once the patient is clinically stable on hormone replacement therapy (45, 53). Nonetheless, such patients should be carefully monitored for additional neurological symptoms given their predisposition toward developing other irAEs, such as severe CNS immune attack (58).

Studies have found that irAEs were associated with a better clinical response (7) and that the development of IH positively predicted survival in patients receiving ICIs (14). Subsequently, there is a concern that the immunosuppressive effect of high-dose GCSs could negatively affect the antitumor efficacy of ICIs, with a recently published study being the first to demonstrate a potential negative effect of high-dose GCSs on ICI treatment efficacy (10). Additionally, GCSs should always be used with caution to reduce the potential for short- and long-term complications.

## Immune checkpoint inhibitor-induced thyroid disorders

### Definition

ICI-induced TDs include thyroid dysfunction, such as overt or subclinical hypothyroidism and thyrotoxicosis, predominantly related to silent (or destructive) thyroiditis and rarely to Graves’ disease (GD), associated with ICI use.

### Epidemiology

The thyroid gland is the endocrine gland most frequently affected by irAEs associated with ICIs. According to recent systematic reviews and meta-analyses, the incidence of TDs depends on the class of ICIs used (1, 21). During anti-PD-1 treatment, thyroid involvement of any grade was one of the most common organ-specific irAEs (59). Different TDs had been observed in approximately 7% of patients receiving ipilimumab, 19% of those receiving PD-1 and PD-L1 inhibitors, and 28% of those receiving combination therapy with ipilimumab and nivolumab (21, 48). Furthermore, some authors had reported even higher frequencies of TDs, involving up to a half of the patients on combination therapy (60).

Table 1 contains data on incidences of TDs during different ICIs treatment regimens collected from recent meta-analyses (1, 18, 20, 21, 22, 23).

#### Hypothyroidism and thyrotoxicosis

The most common thyroid dysfunction is hypothyroidism followed by thyrotoxicosis (or hyperthyroidism). Incidences of hypothyroidism were lower with the anti-CTLA-4 antibody (2.5%-5.2%) than with anti-PD-1/anti-PD-L1 (3.9%-8.5%), while combination therapy was associated with the highest estimated incidence (10.2%-16.4%). Similarly, for thyrotoxicosis differences according to the class of ICIs had been reported, with ipilimumab having low frequencies (0.2%–1.7%), anti-PD-1/anti-PD-L1 drugs having higher frequencies (0.6%–3.7%), and combination therapy having the highest frequency (8.0%–11.1%) (1, 18, 20, 21, 22, 23). Moreover the risk for thyrotoxicosis was significantly greater with anti-PD-1 antibodies than with anti-PD-L1 antibodies and differences among anti-PD-1 drugs were also observed, with nivolumab having lower risk for hyperthyroidism than pembrolizumab (1, 21).

Lower reported frequencies of thyrotoxicosis compared to hypothyroidism at least in part could have been a result of underdetection of mild, asymptomatic, and transient cases before routine monitoring was implemented.

Most of the ICI-related thyroid complications are either mild or moderate (grade 1 or 2, according to CTCAE, version 5.0). High-grade complications (grade 3 or 4) are rare and found mostly during combination therapy (up to 1.3% for thyrotoxicosis) (1, 5, 18).

#### Graves’ disease

ICI-induced GD has rarely been described, with only a few case reports on the same (2). However, clinical trials have found that several patients with GD could have been classified as hyperthyroid; hence, the real incidence of GD might have been underestimated.

### Pathogenesis

The precise mechanisms involved in thyroid gland AEs have yet to be completely understood. It remains unclear whether the presence of thyroid autoantibodies cause thyroid dysfunction or whether they arise as a result of immunological response to thyroid antigens released during destructive thyroiditis (61). Data concerning the association between thyroid irAEs and thyroid autoantibodies have been inconsistent. Indeed, data have shown that patients with elevated thyroglobulin antibodies (TgAbs) and/or thyroid peroxidase antibodies (TPOAbs) at baseline were more likely to develop overt thyroid disease or more severe hypothyroidism following ICI treatment. The titer of thyroid antibodies may be used to identify high-risk patients (61, 62, 63). However, many patients with ICI-induced thyroiditis were negative for TPOAbs and TgAbs, which might suggest a pathogenesis that differs from the classical autoimmune spontaneous thyroiditis (64). It seems that antithyroid antibodies are not pathogenic in ICI-induced thyroiditis and they can not be used to exclude or confirm the disease.

Histologic evaluation of autoimmune thyroiditis (Hashimoto’s and painless thyroiditis) shows lymphocytic infiltration by both B cells and cytotoxic T cells. Inhibiting PD-1, which is expressed by T and B lymphocytes and NK cells, causes proliferation of these cells and more frequent thyroidopathies than CTLA-4 inhibitors, which induce only T-lymphocyte proliferation (48). However, given that thyroidectomies in patients with ICI-induced thyroiditis have been exceptional, little is known about its histologic appearance. Recently Neppl *et al.* described the characteristics of a thyroidectomy specimen from a patient with TRAb-negative nivolumab-induced thyrotoxicosis (65). Accordingly, they observed chronic thyroid lymphocytic inflammation with the formation of granulomas and destruction of follicles, which was distinct from autoimmune TDs, such as Graves’ and Hashimoto’s diseases. However, a predominance of CD8+ T cells within the inflammatory infiltrates, similar to autoimmune thyroiditis, was also observed. Immunohistochemistry showed that PD-L1 overexpression was present especially in areas of follicular destruction. Angell *et al.* reported a case of ICI-induced thyroiditis with unique cytopathologic features, including abundant clusters of necrotic cells, lymphocytes, and CD163-positive histiocytes (66).

### Risk factors

As stated previously, the type of ICI used is strongly correlated with the risk of TDs. However, the dose of ICI and the tumor type were not significantly associated with the incidence of hypo- and hyperthyroidism (1). Preexisting autoimmune disorders, the presence of anti-thyroid antibodies, and increased ^18^FDG uptake in the thyroid before ICI treatment could increase risk for thyroid complications during ICI treatment (61, 62, 63). ICI-induced TDs are more frequent among women and younger patients, which is consistent with the incidence rates observed in the general population (48, 67).

### Clinical presentation and diagnosis

#### Thyroid disorder onset

A retrospective review of clinical trials using ipilimumab showed that primary hypothyroidism occurred from 5 months to 3 years after treatment initiation (68). The onset of thyroid dysfunction in patients receiving anti-PD-1 antibodies has been reported to occur as early as 3 weeks after treatment initiation and up to 10 months following therapy, with a median time to onset of approximately 6 weeks (61, 69). The time to onset of thyrotoxicosis is short in most cases, especially during combination therapy (up to 3 weeks). On the other hand hypothyroidism develops later, with the time to onset of approximately 9 weeks (70). That observation reflects the natural course of ICI-induced thyroiditis.

#### Clinical characteristics

Typically, ICI-induced thyroiditis has a bi- or tri-phasic course with brief and mild or asymptomatic thyrotoxicosis followed by an euthyroid state, which can further progress to hypothyroidism as frequently observed (1, 33). Such a clinical pattern is a result of the initial release of thyroid hormones due to destructive thyroiditis and a subsequent damage leading to hypothyroidism (33). Although frequently asymptomatic, in some patients thyrotoxicosis may cause tachycardia, palpitations, tremors, heat intolerance, sweating, fatigue, and weight loss, which usually resolve spontaneously or progress to overt hypothyroidism in 4–6 weeks (56, 71). The most common symptoms of hypothyroidism are fatigue, constipation, cold intolerance, swelling, and weight gain. Hypothyroidism, in most cases, remains permanent, requiring long-term levothyroxine replacement (6, 33). Although the majority of the cases had been classified as grade 1 or 2 irAEs, serious complications of hypothyroidism, for example, myxedema crisis (72) or myopathy (73) have been rarely reported. Hypothyroidism-associated myopathy was characterized by severe myalgias, arthralgias, and significant creatine kinase elevation, which could be reversed by levothyroxine replacement therapy.

Although prolonged and severe thyrotoxicosis has rarely been observed, cases of ICI-related thyroid storm have been reported (74, 75). Accordingly, patients who demonstrate fever, severe tachycardia or atrial fibrillation, diarrhea, signs of congestive heart failure, need prompt hormonal evaluation and treatment considering the high morbidity and mortality rates among those with thyroid storm (76).

Prolonged symptomatic thyrotoxicosis, sometimes accompanied by ophthalmopathy, might be an indication of ICI-induced GD (77, 78).

Several case reports have described rare ophthalmic side effects including Graves’ ophthalmopathy or a condition resembling such (2, 50, 77, 79, 80, 81). Signs and symptoms typical of Graves’ and ICI-induced ophthalmopathy include proptosis, eye pain, conjunctival redness, periorbital edema, ophthalmoplegia, and swelling of extraocular muscles on MRI. In the latter, however, euthyroidism and negative thyroid-stimulating hormone receptor antibodies (TRAbs) were observed, although increased TPOAbs and TgAbs might be present. Thus, a distinct inflammatory condition called thyroid eye disease (TED)-like orbital inflammatory syndrome (80) or inflammatory orbitopathy (79) can be suspected.

#### Diagnostic procedures

Hormonal test results show suppressed TSH level with elevated fT4 and/or fT3 levels in overt thyrotoxicosis (hyperthyroidism) and elevated TSH level with a decreased fT4 concentration in hypothyroidism. Subclinical thyroid irAEs (normal fT4 and fT3 levels with elevated or decreased TSH levels) are prevalent and might be detected only with regular screening during ICI treatment (63). Close monitoring of hormone levels, at baseline and especially during the first months of treatment, is also necessary for the early detection of the rapid development of significant thyroid irAEs. This is also essential for patients with preexisting Hashimoto’s disease given that they could be more likely to experience ICI-induced thyroiditis and thus considerable changes in their hormonal status (82).

It is important to assess together TSH and fT4 concentrations, especially in asymptomatic patients, because it helps to differentiate thyrotoxicosis (low TSH, high fT4 levels) from central hypothyroidism (low or normal TSH with low fT4 levels). In latter cases IH could be suspected and prompt evaluation of the pituitary–adrenal axis with morning ACTH and cortisol concentrations is necessary. In some patients, suppressed TSH levels may result from high doses of exogenous GCSs for other irAEs or brain metastases.

Once TD is confirmed, thyroid autoantibodies should be measured. In selected cases, such as patients with overt thyrotoxicosis, thyroid ultrasound and scintigraphy might be useful for the differential diagnosis (Fig. 2).

On examination, many patients with thyroiditis exhibit diffuse, non-tender thyromegaly. On ultrasonography, diffuse enlargement of the thyroid gland, decreased internal blood flow, and low internal echogenicity could be observed in ICI-induced destructive thyroiditis (56). Typically, negative TRAbs, reduced or absent tracer uptake on technetium or iodine scanning, and/or increased 18-fluorodeoxyglucose uptake on PET (^18^FDG-PET) have been used to confirm a diagnosis of destructive thyroiditis (21, 56). Patients with increased ^18^FDG uptake in the thyroid before ICI-treatment could have an increased risk for developing/worsening TDs (61). Higher ^18^FDG uptake could be correlated with the severity of hypothyroidism and TgAb positivity (63) and might predict the development of thyroiditis with subsequent hypothyroidism (83).

In contrast, GD is characterized by high blood flow on Doppler ultrasound. Once GD is suspected, TRAb testing and thyroid scintigraphy should be performed. Unlike destructive thyroiditis or toxic nodular goiter, high titers of TRAbs and high diffuse homogeneous iodine uptake in the thyroid gland is expected in GD. Diagnostic problems could be found in patients previously exposed to iodinated contrast agents for CT (CT), which could lower the thyroid’s iodine uptake (33, 50).

Monitoring patients receiving ICI is recommended with TSH and fT4 assessment before the first dose and then at least once a month for the first 6 months (Fig. 1). When results are normal without signs and symptoms of thyroid dysfunction, the aforementioned laboratory tests can be performed every 3 months for another 6 months and every 6 months thereafter. Each symptomatic patient should be further examined (33).

### Management

In mild, transient, and asymptomatic cases of thyroid dysfunction, such as subclinical hypothyroidism with TSH levels lower than 10 mIU/L, only careful observation with repeated testing may be needed. However, treatment should be discussed in cases with TSH levels between 5 and 10 mIU/L associated with either clinical symptoms or the presence of TPOAbs (45). For the treatment of overt hypothyroidism, levothyroxine administration is frequently started at a low dose of 25–50 μg/day but it could be as well initiated with the full estimated replacement dose of 1.6 µg/kg of body weight, especially in young and otherwise healthy individuals. Lower initial doses (12.5–25 μg/day) and slow modifications are particularly important in elderly patients or patients with cardiac diseases (33, 48). Mildly elevated TSH levels (5–10 mIU/L) can usually be replaced with low doses of levothyroxine (25–50 μg/day) (84). Initial dose should be adjusted according to serum TSH levels every 4 to 6 weeks at the beginning and every 3 months thereafter. AI should be ruled out before starting levothyroxine to prevent triggering potential adrenal crisis. Attempts to progressively withdraw levothyroxine can be made at the end of ICI treatment, especially if suppression of TSH level on stable dose of levothyroxine is observed, with continued clinical and TSH monitoring every 4 to 6 weeks.

Symptomatic thyrotoxicosis is routinely treated using beta-adrenoceptor blockers, such as propranolol, to alleviate palpitations. However, high-dose GCSs might be indicated in severe cases of thyrotoxicosis due to thyroiditis. Initiation of anti-thyroid drugs, such as methimazole (thiamazole) or propylthiouracil, should only be considered in rare cases of Graves’ hyperthyroidism given that these agents are not effective in destructive thyroiditis (33, 56). Radioactive iodine could be used in selected patients with GD, for example, those intolerant of oral treatment (48). Patients in a thyrotoxic state due to thyroid irAEs should be further monitored at least every 2 to 3 weeks (33).

Patients with Graves’ orbitopathy often benefit from high-dose GCS treatment (77). Similarly, such treatment was helpful in resolving symptoms in most cases with TED (80), although prolonged treatment may occasionally be needed (81).

Both preexisting and new ICI-induced thyroid diseases are not contraindications for the initiation or continuation of cancer immunotherapy. However, immunotherapy should be withheld in patients with severe hypothyroidism or thyrotoxicosis until their general condition and laboratory results improve with appropriate endocrine therapy. In cases of orbitopathy, immunotherapy should be stopped and carefully reintroduced after individual assessment (45) but should be discontinued in severe or refractory cases (80, 81).

## Immune checkpoint inhibitor-related diabetes mellitus

### Definition

ICI-related DM is defined as new-onset insulin-dependent DM following ICI treatment for a malignancy. The presence of preexisting type 2 DM (T2DM), which has a different pathogenesis, does not preclude the development of ICI-induced DM.

### Epidemiology

Albeit rare, ICI-related DM is a potentially life-threatening complication of ICI therapy. The first reports of ICI-induced DM had been published in 2015 (85, 86). Although new-onset DM had not been reported in clinical trials involving CTLA-4 inhibitors, it had been reported in <1% of patients included clinical trials on PD-1 inhibitors, with higher rates (up to 1.5%) being observed with the combined use of CTLA-4 and PD-1 inhibitors (87). The latest review identified 91 case reports of ICI-related DM from the literature (88). Many cases of DM following ICI therapy were reported in 2016 and 2017, after anti-PD-1 antibodies had been approved in 2014 (2). Prior to this date, DM following ICI therapy was considered to be very rare and was not at all reported in a 2016 meta-analysis (17). Moreover, only 13 cases (0.2%) had been reported in a 2018 meta-analysis of 38 clinical trials including 7551 patients (1). A recent study showed that among 1444 patients receiving ICIs over 6 years at the Mayo Clinic, 1.4% developed new-onset insulin-dependent DM or significant unexplained worsening of T2DM. Among 1163 patients receiving PD-1 inhibitors, 1.8% satisfied the criteria for ICI-induced DM, whereas none of the 281 patients receiving the CTLA-4 inhibitor ipilimumab developed DM (89). To date, the majority of ICI-induced DM cases have been associated with the use of PD-1 inhibitor therapy (90). ICI-related DM appears to be extremely rare with anti-CTLA-4 monotherapy (Table 1). Importantly, most of the cases with ICI-induced DM on ipilimumab received anti-PD-1 and/or interferon pretreatment (91, 92). The main tumor types among patients who developed DM included melanoma (53%) and NSCLC (15%) (88).

### Pathogenesis

The PD-1/PD-L1 pathway is a key regulator in T-cell activation and tolerance. The role of ICIs in the pathophysiology of type 1 DM (T1DM) had been investigated in both mice and humans. Non-obese diabetic (NOD) mice develop rapid-onset diabetes following the blockade of PD-1 or PD-L1 but not PD-L2 (93). This fact corresponds with the finding that pancreatic islets express PD-L1 at low levels in mice, which is dramatically up-regulated in inflamed islets (93, 94). In humans, polymorphisms in the CTLA-4 and PD-1 gene have been correlated with increased susceptibility to a variety of autoimmune disorders, including T1DM (95, 96). There was no evidence of CTLA-4 expression on pancreatic islets, although the transgenic over-expression of anti-CTLA-4 Fv on β cells could protect NOD mice from autoimmune diabetes (97). The CTLA-4 and PD-1/PD-L1 pathways have also been studied in T cells from patients with ‘classic’ T1DM. A few studies have reported decreased PD-1 expression on activated T cells in patients with T1DM (98, 99). These observations suggest that an imbalance between activated and resting T cells might promote autoimmunity via a mechanism similar to that of PD-1 blockade therapy. More recently, Granados *et al.* demonstrated further PD-1 dysregulation as activated peripheral T cells from children with new-onset T1DM failed to upregulate PD-1 upon T-cell receptor stimulation (100). Regulatory T cells (Tregs) express both CTLA-4 (101) and PD-1 (102), which are essential in their activation and suppressive role in peripheral immune tolerance (103), and a deficiency in the ability of Tregs to up-regulate PD-1 and efficiently use the PD-1/PD-L pathway has been observed in patients with T1DM (104). Furthermore, human pancreatic β cells express PD-L1, which is induced by IFN-γ. This expression is upregulated in inflamed islets and is associated with CD8+ T cell infiltration (105, 106). Although specific CTLA-4, PD-1, and PD-L1 polymorphisms have been associated with increased susceptibility for developing T1DM, these polymorphisms have not been studied in patients who have developed ICI-related DM.

### Risk factors

Predisposing factors for ICI-induced DM have not been fully identified. However, genetic predisposition may contribute to development of ICI-induced DM as it does to T1DM. Although HLA (HLA) class II alleles at 6p21 account for up to 30%–50% of the genetic T1DM risk, more than 40 non-HLA susceptibility gene markers have been also confirmed. These include insulin, PTPN22, CTLA4, IL2RA, IFIH1, and other recently discovered loci (107). Differences between populations and diabetic genotypes do exist given that DR3-DQ2 and DR4-DQ8 haplotypes are major risk factors for T1DM, still conferred by each of these alleles on their own (107), and that DR4-DQ4 and DR9-DQ9 haplotypes have been linked to fulminant DM among Asians (108). HLA typing was performed in a subset of the published ICI-induced DM cases and patterns in HLA susceptibility have emerged as well (Table 2). High prevalence of either DR4 or DR3 in Caucasian patients have been noticed. Of 51 patients with ICI-induced DM, 31 (61%) had susceptibility genotypes for T1DM or fulminant DM, whereas 2 (4%) had a protective genotype (88). Another study showed that HLA-DR4 was found in 76% of patients, a significantly higher frequency than that in US Caucasians or even patients with spontaneous T1DM (21). Although HLA-A2 was also frequently detected (59% of patients), its detection frequency did not significantly differ from that reported among US Caucasians (47.4%). HLA-DR3, which is also increased among patients with T1DM (34.1%), was detected at a similar frequency among patients with ICI-related DM (35%, 6/17). HLA-DQ8 (DQB1*0302) was found in 38% of the patients, a frequency similar to that for patients with T1DM (21). Interestingly, in this study none of the subjects expressed the T1DM protective allele HLA-DR2 (109).

**Table 2:** HLA genotypes and islet autoantibodies in patients with ICI-induced DM in recent studies.

Study	de Filette *et al.* (21)	Stamatouli *et al.* (109)	Kotwal *et al.* (89)
No. of patients	91	27	21
Cancer type			
Melanoma (%)	53	52	45
Lung (%)	15	18	25
Other (%)	32	30	30
HLA type			
Subject with full HLA typing (*n*)	51	23	
Susceptible HLA (%)	61	69	–
	A2, DR3, DR4, DR949	DR4-DQ8, DR3-DQ2 or DR4-DQ40	
Protective & susceptible HLA (%)	4	0	–
Protective HLA (%)	16	0	–
HLA-DR4 (%)	49	76	–
Positive islet autoantibody			
Subjects with autoantibody testing (*n*)	88	25	7
Any islet autoantibody (%)	53	40	71
Two or more islet autoantibodies (%)	15	21	–
Anti-GAD65 autoantibody (%)	51	36	57

anti-GAD65, anti-glutamic acid decarboxylase antibody; DM, diabetes mellitus; ICI, Immune checkpoint inhibitor.

### Clinical presentation and diagnosis

#### Diabetes mellitus onset

The onset of ICI-related DM ranges from a few weeks, sometimes even after the first or second cycle of immunotherapy (109, 110), up to more than a year after the initiation of immunotherapy (109, 111). On average, it was diagnosed after 4.5 cycles, although an earlier onset was observed during combination immunotherapy (after 2.7 cycles), as well as during anti-PD-1/PD-L1 monotherapy (88).

#### Clinical characteristics

ICI-induced DM shares many similarities, as well as differences, with autoimmune T1DM. Patients with ICI-induced DM were older at diagnosis compared to those with T1DM, with a median age at ICI initiation of 60 years and male predominance (60%) which probably reflects the demographics of patients receiving ICIs (88, 89). In most cases, the clinical presentation showed a severe course. Patients with ICI-related DM often present with signs and symptoms of hyperglycemia (polyuria, polydipsia, and weight loss) or diabetic ketoacidosis (DKA) (nausea, vomiting, abdominal pain, hyperventilation, lethargy, or coma) (Fig. 1). DKA was present in the majority of the reported cases (66.7%–71%), with a high median presenting glycemia of 565 mg/dL (range 209–1211) and a rather low glycated hemoglobin level of 7.6% (range 5.4–11.4) (88, 89). Noteworthily, publication bias may exist toward severe presentations due to an underrepresentation of milder diabetes cases in the literature. This type of DM shares many clinical features with fulminant DM, a subtype of T1DM first described in Japan (112). According to the 2012 revised criteria of the Japan Diabetes Society (113), a diagnosis of fulminant DM is confirmed when all of the following three findings are present: (1) rapid occurrence of diabetic ketosis or DKA (~7 days) after the onset of hyperglycemic symptoms, (2) plasma glucose ≥ 288 mg/dL and HbA1c < 8.5% at the first visit, and (3) urinary C-peptide excretion <10 μg/day or fasting serum C-peptide level <0.3 ng/mL and serum C-peptide <0.5 ng/mL after intravenous glucagon administration (or after a meal) at onset. Low or undetectable C-peptide levels were present at diagnosis in 58 out of 69 cases (84%) with new-onset ICI-related DM and in the majority of patients with pre-existing T2DM (89%) who had unexplained worsening of glycemic control (88, 89). Several findings relatively common in many cases of fulminant DM do not seem to be common in ICI-related DM. In particular, islet autoantibodies, which were generally undetectable in fulminant DM, were found in half of the case reports reviewed (113). Moreover, elevations in serum pancreatic enzyme levels, which was observed in 98% of patients with fulminant DM, was not common among those with ICI-related DM (113). Elevated lipase levels were detected in 52% and 57% of reported cases of ICI-induced DM (88, 89). However, pancreatic enzyme levels were not routinely reported in the available case reports. Finally, flu-like symptoms were commonly reported in fulmiant DM but not in ICI-related DM.

#### Islet autoantibodies

Of the 88 cases reviewed, 47 (53%) were positive for at least one of the islet autoantibodies, while 13 (15%) were positive for ≥2 islet autoantibodies (88) (Table 2). An antibody against GAD65 was the most common (positive in 51% of the patients). Elevated titers of other T1DM autoantibodies appear to be less prevalent than GAD65 at time of diagnosis. Moreover, 18% of the patients were positive for insulinoma-associated antigen-2, 13% for islet-cell antibodies, 26% for anti-insulin, and 4% for zinc transporter 8 (6, 88). This differs from the ‘classic’ T1DM where autoantibodies are present in 80%–95% of the patients (114, 115). The presence of autoantibodies at the time of diagnosis has previously been suggested to be related to an earlier onset of ICI-induced DM (109, 110, 116, 117). Another hypothesis suggests that a subset of patients who develop ICI-induced DM likely have preexisting islet autoantibodies, which may be an early form of a latent autoimmune diabetes in adults (116, 118, 119). In contrast, rapid-onset ICI-related DM presenting with DKA in response to combination immunotherapy with confirmed GAD65 antibody seroconversion has also been reported (120). These divergent reports suggest that baseline autoimmune DM antibody testing may not be particularly useful as biomarkers for predicting individuals susceptible to ICI-related DM.

#### Pancreatic inflammation

It remains unclear to what extent the pancreatic exocrine gland is involved in the process. Biochemical evidence of exocrine pancreatic inflammation with elevated lipase levels was reported in about 50% of all ICI-related DM cases. Patients with such laboratory findings are often asymptomatic (109, 121). Previous studies have hypothesized that autoimmune T1DM is a combined endocrine–exocrine disease wherein loss of functional β-cell mass is most clinically apparent (122), and non-specific elevations in amylase and lipase occur in 16–25% of cases with DKA in ‘classic’ T1DM (123). In the context of ICI therapy, asymptomatic elevations in lipase and/or amylase have also been reported in the absence of new-onset DM (124, 125). Several authors have also described radiographic changes in pancreatic volume during immunotherapy, notably pancreatic enlargement before DM onset followed by a decrease in volume (121, 126). In the study by Michot *et al.*, 3 of 21 patients (14%) developed clinical or radiologic immune-related pancreatitis that led to permanent treatment discontinuation in 15 of 21 (71%) patients, while the increase in lipase was not considered clinically significant, and treatment was continued (125). Ongoing pancreatic inflammation has been hypothesized to be a factor in the precipitation of DM; however, this is an additional question that should be addressed in prospective studies (109, 127).

It is also still unclear whether the autoimmune destruction in the islet involves other islet cells apart from β-cells. Random glucagon levels were not reduced in most patients, suggesting that α-cells have not been affected. Stamatouli *et al.* found that random glucagon levels were within the normal range in a small sample of four patients in whom glucagon was measured (109). However, Marchand *et al.*, who analyzed data from six subjects with ICI-induced DM, showed that two patients who presented with fulminant DM had a blunted glucagon response during the mixed meal test (MMT) (121). In this study, the pancreatic volume was decreased at diabetes onset in two patients with fulminantDM, while all patients presented a subsequent decrease of pancreas volume during follow-up (121). This aspect still requires careful exploration of pancreatic endocrine α-cell function with MMT and exocrine function to determine whether autoimmune destruction is specific to islet α-cells.

#### Diagnostic procedures

Patients demonstrating signs or symptoms of hyperglycemia should undergo glucose level testing and DKA evaluation using serum or urine ketones while also considering venous blood gas evaluation (Fig. 1). Given the possibility of a rapid increase in blood glucose levels and swift progression to DKA, patients receiving ICIs should be informed of the possibility for developing DM and educated on the symptoms of hyperglycemia and DKA so that they or their family members could immediately contact attending physicians as soon as these symptoms develop. Hyperglycemia may be detected incidentally (in asymptomatic patients), especially given that plasma glucose is obtained as part of routine monitoring for patients receiving ICI therapy (Fig. 1). In such cases, prompt recognition of marked hyperglycemia and treatment with insulin can prevent progression to DKA. Attending oncologists should determine glucose levels upon visitation and consult endocrinologists as soon as possible when blood glucose levels are increased (fasting blood glucose ≥ 126 mg/dL; random blood glucose ≥ 200 mg/dL). C-peptide measurement in the blood or urine may be helpful in assessing endogenous insulin production. Accordingly, low C-peptide levels should prompt high suspicion for ICI-related DM. The presence of preexisting T2DM, which is common in this age range, does not preclude the development of ICI-induced DM. Upon the detection of new-onset DM or worsening glycemia in patients with T2DM, HbA1c and pancreatic autoantibodies (especially GAD65) should be analyzed to support the diagnosis of ICI-related DM. However, the presence of islet autoantibodies, detectable in ~50% of patients, is not an absolute requirement for the diagnosis and treatment of ICI-related DM.

#### Management

Close glycemic monitoring is usually necessary and can lead to prompt diagnosis of ICI-related DM and prevention of DKA.

Given the rapid increase in glycemia and high prevalence of DKA, patients with ICI-induced DM often require admission to hospital for monitoring and treatment. In case of severe DKA admission to intensive care units is obligatory. The management is based on intensive insulin treatment for glucose control, together with supportive measures (i.e. hydration and correction of electrolytes) according to standard guidelines (128). There is currently no effective method for preventing or limiting the onset if this irAE. High-dose GCSs (a standard treatment for non-endocrine irAEs) are not recommended for the treatment of ICI-induced DM given that GCSs do not reverse DM (120, 129, 130). Patients requiring high-dose of GCSs for the treatment of other irAEs should undergo careful blood glucose monitoring.

Restarting ICI treatment should be considered once adequate glucose control has been established. ICI-related DM almost invariably results in long-term need for insulin.

## Immune checkpoint inhibitor-related primary adrenal insufficiency

### Definition

ICI-related PAI is characterized by glucocorticoid and mineralocorticoid deficiency accompanied by elevated plasma ACTH levels.

### Epidemiology

PAI is a very rare complication of ICI therapy and has been reported in a few cases (2, 131). Patients with PAI, 60% of whom were male, had a median age of 52 years. One study showed that among the six patients with PAI, three had metastatic melanoma. Anti-PD-1 antibody monotherapy was used in four of the six patients, while ipilimumab was used in two patients (2).

### Clinical presentation and diagnosis

#### Primary adrenal failure onset

The median onset of PAI symptoms was 10 weeks after ICI initiation (range: 1.5–36) (2).

#### Clinical characteristics

Signs and symptoms of adrenal insufficiency were specified previously. Although hyponatremia and hyperkalemia are common in PAI due to the presence of both GCS and mineralocorticoid deficiency, hypoglycemia and hypercalcemia can be also seen (Fig. 1).

Several approaches can be used for the differential diagnosis of adrenal insufficiency among patients with malignancy on immunotherapy. SAI and PAI should be distinguished from one another given that PAI requires mineralocorticoid replacement in addition to GCS replacement. SAI can be caused by ICI-related hypophysitis or pituitary metastasis, whereas PAI may be due to immunotherapy, bilateral adrenal metastases, or bilateral adrenal hemorrhage. Most significantly, SAI due to hypophysitis is much more common than PAI. Hyponatremia in patients with malignancy can be also secondary to SIADH. This highlights the importance of careful investigation for hyponatremia while excluding hypocortisolemia as a potential life-threatening cause of low plasma sodium.

### Diagnostic procedures

Assessment of ACTH and cortisol levels is recommended when PAI is clinically suspected. However, empiric treatment with HC should not be delayed in acutely ill patients with clinical signs and symptoms of adrenal insufficiency. An elevated ACTH (e.g. greater than twofold the upper limit of the reference range) in a patient with low or low-normal morning cortisol levels (≤5 µg/dL) is consistent with PAI (Fig. 1). Cosyntropin (Synacthen) stimulation testing can also be helpful in the diagnostic workup of adrenal insufficiency. Low plasma aldosterone and elevated renin levels can be helpful in determining mineralocorticoid deficiency consistent with PAI. The utility of measuring adrenal autoantibodies has not been studied in ICI-related PAI. Elevated 21-hydroxylase and adrenal cortex antibody titers were found in patients who developed PAI (132). Whether adrenal autoantibodies play a role in the pathogenesis, prediction, or prognosis of ICI-related PAI still remains unclear. Abdominal imaging in ICI-related PAI may show evidence of adrenalitis, reflected as bilaterally enlarged adrenal glands with relatively smooth borders accompanied by increased ^18^FDG during PET/CT (6). If abdominal imaging results are older than 3 months (performed as part of cancer follow-up), adrenal CT should be performed to look for variations in adrenal morphology suggestive of adrenal inflammation or adrenal atrophy and to eliminate the differential diagnosis of bilateral adrenal metastases, hemorrhage or tuberculosis.

### Management

Stress-dose GCSs should be immediately administered to any patient with known PAI presenting with an acute adrenal crisis or severe illness. HC should also be considered as an empiric treatment among critically ill patients who present with signs or symptoms indicating adrenal insufficiency. A typical stress dose of corticosteroids includes an initial dose of 100 mg HC IV followed by 50 mg HC IV every 6 h. Depending on the clinical course, this regimen can gradually be tapered to basal GCS replacement with 15–25 mg of HC PO daily in divided doses. Mineralocorticoid replacement with fludrocortisone is required for patients with confirmed aldosterone deficiency (Fig. 2). Patients with ICI-related PAI require long-term glucocorticoid and mineralocorticoid replacement (6).

A new concern regarding ICI-related PAI among patients with melanoma has been raised. One study has hypothesized that melanoma patients who suffer from Addison’s disease are at higher risk for recurrence or an unfavorable disease course due to elevated levels of ACTH and α-melanocyte-stimulating hormone (α-MSH) (133). On the contrary, melanoma patients with adrenal insufficiency due to ipilimumab-induced hypophysitis had higher survival rates (14). It is speculated that low ACTH and α-MSH levels exert a protective role in patients with persistent SAI. ACTH and the different isoforms of MSH can bind to melanocortin receptors, some of which (e.g. MC1R) are overexpressed in human melanoma cells. Thus, the elevated ACTH levels and its cleavage product α-MSH typical for PAI may promote receptor activation and increased proliferation, placing patients with melanoma who have ICI-induced PAI at higher risk for recurrence or an unfavorable disease course. Nonetheless, this hypothesis needs further study given that no data are currently available on the affinity of the melanocortin receptors for ACTH and the MSH subtypes in the context of coexisting melanoma and ICP-induced PAI.

## Immune checkpoint inhibitor-related primary hypoparathyroidism

Single cases of primary hypoparathyroidism presenting with severe hypocalcemia during ICI therapy have been reported. Accordingly, two case reports involving patients with melanoma who had severe hypocalcemia with undetectable plasma PTH associated with nivolumab/ipilimumab treatment (134, 135) and another involving a patient with small-cell lung cancer on nivolumab treatment (136) have been published. In the latter case, the presence of activating autoantibodies against the calcium-sensing receptor (CaSR) was confirmed. In all but one case, parathyroid function did not recover during follow-up and required continued calcium and active vitamin D treatment for hypocalcemia (Figs 1 and 2).

## Acquired generalized lipodystrophy

Lipodystrophy has never been previously described as an irAE related to ICIs. However, three case reports of acquired generalized lipodystrophy characterized by loss of subcutaneous fat tissue, central obesity, and insulin resistance with decreased leptin level during ICI treatment have recently been published. Worsening of glycemic control was perhaps mostly related to increased insulin resistance given that it was accompanied by the progression of the lipodystrophy (137, 138, 139).

## Autoimmune polyendocrine syndrome

Only a few cases of ICI-related autoimmune polyendocrine syndrome (APS) have been reported thus far. Most cases, where PAI typically occurred with APS-2, were treated with anti-PD-1 or anti-PD-L1 antibodies (26, 132, 140). HLA typing revealed haplotypes, which were associated with increased susceptibility to ‘classic’ T1DM and APS-2 (141). A case report on APS-2, characterized by PAI and T1DM and accompanied by hypophysitis, in a 60-year-old male patient receiving atezolizumab had been published. The patient was positive for 21-hydroxylase and pituitary antibodies and negative for islet cells antibodies (26). In a case report by Gunjur *et al.*, a 78 year-old female receiving pembrolizumab presented with a typical APS-2 triad of PAI, T1DM, and hypothyroidism (140). The patient was positive for anti-islet antibodies. Another report by Paepegaey *et al.* showed that a 55-year-old female positive for 21-hydroxylase antibodies who was on pembrolizumab for a metastatic melanoma developed PAI and hypothyroidism (132). Strikingly, the age upon diagnosis of APS-2 was much higher among patients receiving ICI than among those with spontaneous APS-2 (30–40 years old on average) which probably may reflect the population receiving ICI therapy. Patients with spontaneous APS-2 showed a similar pattern of HLA class II alleles to those with spontaneous T1DM (with significantly higher representation of HLA-DR3 and DR4 serotypes than matched health controls). This appears to be similar to that for ICI-induced DM and APS-2 (Table 3). Thus, HLA class II haplotype may appear to serve as a useful biomarker for predicting risk of endocrine irAEs (142).

**Table 3:** HLA genotypes and autoantibodies in patients with ICI-induced APS-2 (at least two components) in recent studies.

Study	Gunjur *et al.* (140)	Lanzolla *et al.* (26)	Paepegaey *et al.* (132)	Sakurai *et al.* (142)	Mellati *et al.* (141)
Cancer type	Melanoma	NSCLC	Melanoma	RCC	SCC jaw
Therapy	Anti-PD-1	Anti-PD-L1	Anti-PD-1	Anti-PD-1	Anti-PD-1
Age (years)	78	60	55	68	66
Sex (F/M)	F	M	F	F	F
Endocrine irAEs	TD	T1DM	TD	TD	TD
	T1DM	Hypoadrenalism	Hypoadrenalism	T1DM	T1DM
	Hypoadrenalism	Hypophysitis			
HLA type	DRB1*04	DRB1*04	–	DRB1*09	DR3-DQ2
	DQB1*02	DQB1*03		DQB1*03	DR4-DQ8
	DQA1*01				
Positive autoantibodies	Anti-GAD65	Anti-21OH	Anti-21OH	Anti-TPO	Anti-TPO
	Anti-IA2	APA		Anti-Tg	Anti-GAD65

Anti-21OH, anti-21 hydroxylase autoantibodies; anti-GAD65, anti-glutamic acid decarboxylase antibody; anti-IA2, anti-islet antibody; anti-TG, anti-thyroglobulin antibodies; anti-TPO, anti-thyroid peroxidase antibodies; APA, anti-pituitary autoantibodies; NSCLC, non-small-cell lung cancer; PAI, primary adrenal failure; RCC, renal cell carcinoma; T1DM, type 1 diabetes mellitus; TD, thyroid disease.

## Conclusions

Considering that endocrine irAEs can lead to life-threatening consequences, such as an adrenal crisis, thyroid storm, severe hypocalcemia, and DKA, endocrinologists and oncologists should be aware of the clinical features of ICI-induced endocrinopathies. Moreover, multidisciplinary cooperation is necessary to improve the prognosis of patients with cancer. Although advance identification of patients at greatest risk for developing ICI-induced endocrine irAEs would be beneficial, further studies are still required.

## Declaration of interest

M S B received lecture honoraria from Bristol-Myers Squibb and Roche. I C-O has nothing to disclose.

## Funding

This work did not receive any specific grant from funding agencies in the public, commercial or not-for-profit-sector.

## Author contribution statement

Both authors planned the concept of this review, wrote and revised the final manuscript.
